# Aldehyde group driven aggregation-induced enhanced emission in naphthalimides and its application for ultradetection of hydrazine on multiple platforms†
†Electronic supplementary information (ESI) available: Materials & methods, experimental procedures, comparison table, UV-vis spectra, DLS spectra, IR spectra, cell imaging data in wash-free media and spectroscopic characterization data (multinuclear NMR and HRMS) (Fig. S1–S9). See DOI: 10.1039/c8sc00643a


**DOI:** 10.1039/c8sc00643a

**Published:** 2018-04-06

**Authors:** Niranjan Meher, Swagatika Panda, Sachin Kumar, Parameswar Krishnan Iyer

**Affiliations:** a Department of Chemistry , Indian Institute of Technology Guwahati , Guwahati-781039 , Assam , India; b Department of Bioscience and Bioengineering , Indian Institute of Technology Guwahati , Guwahati-781039 , Assam , India; c Centre for Nanotechnology , Indian Institute of Technology Guwahati , Guwahati-781039 , Assam , India . Email: pki@iitg.ernet.in ; Fax: +91 361 258 2349

## Abstract

Restriction of intramolecular motion (RIM) of rigid aromatic cores is the most universal mechanism so far that can successfully explain almost all AIE/AIEEgenic systems. By designing two novel naphthalimide derivatives (**NIB** and **NIBD**), we experimentally demonstrated the effect of a single formyl group that can efficiently transform an aggregation caused quenching (ACQ) system (**NIB**) into an AIEEgenic system (**NIBD**) by strengthening the RIM process. Besides, the newly designed naphthalimide AIEEgen (**NIBD**) accomplished ultrasensitive detection of hydrazine at the parts per trillion level (LOD/81 ppt) in aqueous media with high selectivity and enormous improvement over the existing state of the art. An exceptional sensitivity is also achieved in the vapor phase (LOD/0.003%) using a Whatman paper strip based portable device for simple and cost-effective on-site detection. The detection mechanism involved a reaction-based spontaneous formation of a non-fluorescent hydrazone Schiff base derivative (**NIBDH**). The *in vitro* potentiality of the AIEEgenic probe was also demonstrated in two mammalian cell lines *i.e.* HeLa (human cervical cancer cell line) and HEK293T (Human embryonic kidney cell line that expresses a mutant version of the SV40 large T antigen). Owing to the highly selective formation of the hydrazone Schiff base complex with hydrazine, **NIBD** responds to the existence of hydrazine in both these cell lines without any interference from other biologically rich metal ions and amino acids. These outcomes could initiate a much wider use of formyl group induced condensed state emission and a key hypothesis that could generate newer avenues for ACQ to AIEE transformations for several practical applications including hydrazone Schiff base complexation for probing and manipulating hydrazine biology associated with several metabolic activities.

## Abstract 2

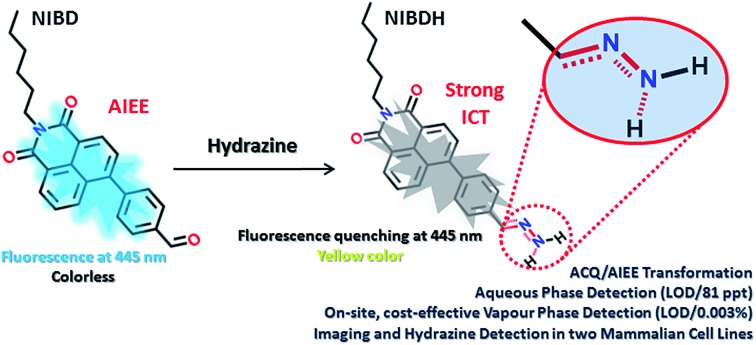
A new concept of formyl group induced ACQ to AIEE transformation is established in naphthalimide congeners. Also, ultradetection of hydrazine *via* Schiff base complexation over multiple platforms is presented.

## Introduction

**Fig. 1:**
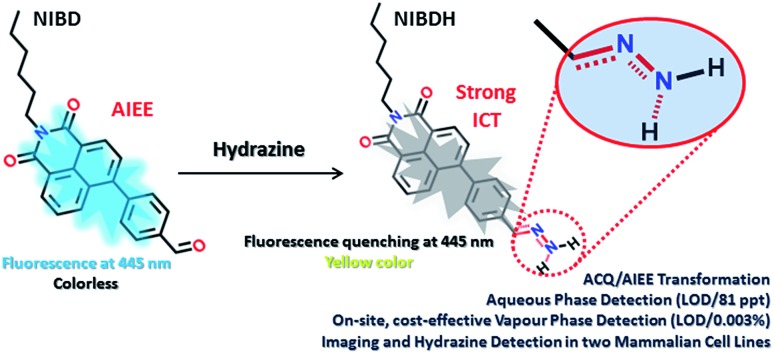
Proposed sensing mechanism of hydrazine on multiple platforms *via* non-fluorescent hydrazone Schiff base complexation.

Organic molecules with condensed state emission are receiving huge attention due to their versatile real-world applications.1a,b After the discovery of hexaphenyl silole (HPS) in 2001, a number of aggregation induced emission (AIE) and aggregation induced enhanced emission (AIEE) active cores have been reported by various groups. In 2014, through a review article by Tang *et al.*, a unified RIM process as the basic working mechanism for all luminogens was proposed.1*a* In the solution state, the dynamic intramolecular motion of the rigid aromatic cores consumes the maximum portion of the excitation energy that makes the molecules non-emissive or weakly emissive. The formation of nanoaggregates in poor solvent interlocks the molecules and stops the dynamic motion and the system becomes fluorescent. However, in this instant as a rare observation, the formyl group was found to induce the aggregated state emission of naphthalimides and this vital hypothesis could find newer avenues to transform the ACQ systems into AIE/AIEE systems. Moreover, the formyl group containing the naphthalimide AIEEgen was perceived to react spontaneously with hydrazine to form Schiff base derivatives and has been applied to detect hydrazine in multiple challenging platforms (Fig. 1).

Regardless of being a strong human carcinogen and having a threshold limit value (TLV) of 10 ppb as identified by the US-EPA, hydrazine is extensively used in several industrial applications as a chief chemical reagent including pesticides, pharmaceuticals, textiles, dyes, and many more.1c–f Hydrazine has mutagenic effects and can harm the human central nervous system, kidneys, lungs and liver.^2^ It is also used as a propellant in projectile propulsion systems and gas precursor for air bags due to its greater enthalpy of burning.3*a* Reports have witnessed that more than 120 000 tons of approximately 64% hydrazine hydrate solution were produced globally per year.3*b* Due to its high solubility in water, hydrazine readily contaminates the soil and ground water during manufacturing, usage, transport, disposal, *etc.*, and could enter the food chain easily.

**Fig. 2:**
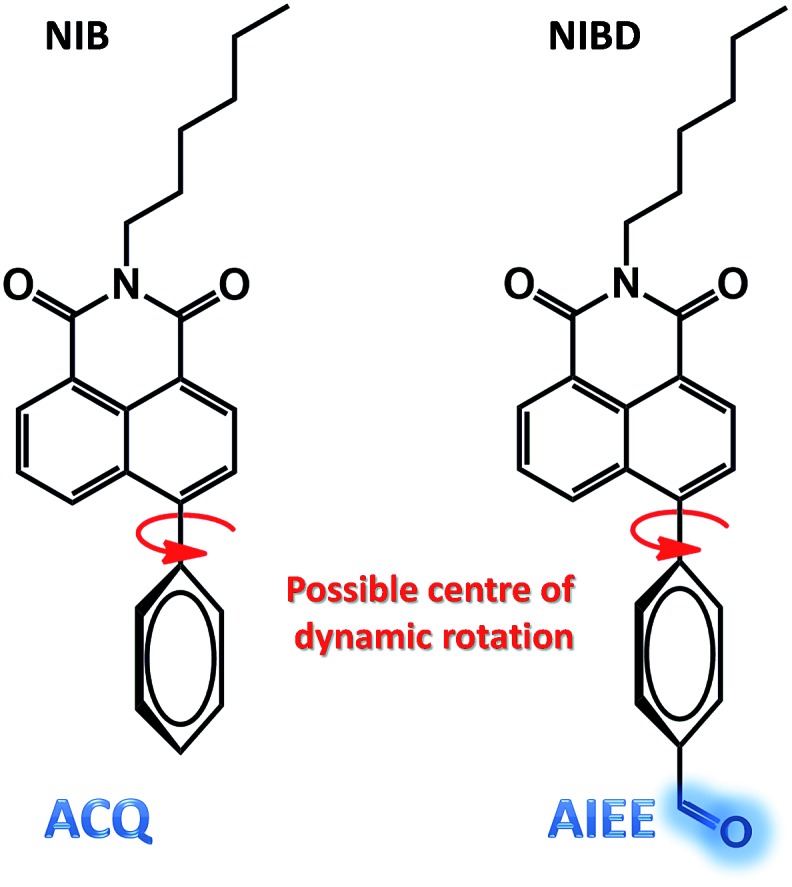
Chemical structure of **NIB** and **NIBD** representing the possible centre for dynamic rotation and demonstrating the formyl group induced aggregation induced enhanced emission.

Among the detection methods developed for hydrazine, electrochemical and chromatographic techniques are time consuming and suffer primarily from portability and on-site application issues that make them very expensive, complex and impractical.^4^ Fluorescence signalling based sensing of hydrazine is receiving increased attention owing to its high selectivity and sensitivity and because it can be applied in both liquid and solid state. In this respect, few molecular probes specific for hydrazine have been designed.^5^,^6^ However, to the best of our knowledge, almost all the classical fluorometry based detections were executed in mixed solvent systems (organic and aqueous), which could be the primary reason for their poor detection limit (Table S1†). Again, sensitive detection of hydrazine vapor using a solid support is another crucial hurdle associated with the existing fluorophores.^6^ Herein, we report two novel naphthalimide congeners, through which we could demonstrate a new hypothesis that incorporation of a formyl group into an ACQ molecule (**NIB**) can induce AIEE characteristics to it (**NIBD**) leading to unprecedented photophysical properties (Fig. 2). Besides, **NIBD** exhibited highly selective ultradetection of hydrazine at the ppt level in aqueous medium for the first time. More importantly, the formation of the non-fluorescent hydrazone Schiff base derivative appears to be highly effective in the vapor phase with huge advancement from the existing state of the art and could be applied for instant on-site testing (see ESI, Table S1†). The *in vitro* potential of the probe was also demonstrated in two different mammalian cell lines with a high signal-to-noise ratio.

## Results and discussion

### Design, synthesis and characterization of naphthalimides with ACQ and AIEE properties

Both the naphthalimides were synthesized by alkylation of 4-bromo-1,8-naphthalicanhydride followed by Suzuki coupling with phenylboronic acid/4-formylphenylboronic acid in good yields (see ESI, Scheme S1†). The synthesized molecules were well characterized by multinuclear NMR spectroscopy and HRMS. The complete synthetic processes and associated spectra have been presented in the ESI.† The phenyl group was intentionally incorporated onto the naphthalimide ring to implement the restriction in intramolecular rotation (RIR) phenomenon in their condensed state, whereas the formyl group was introduced to provide a specific recognition site for hydrazine through the favourable hydrazone Schiff base formation at room temperature. However, the exceptional effect of the formyl group on inducing aggregated state emission was explored through a comparative analysis of **NIB** and **NIBD** in their condensed state (Fig. 2).

### Aggregation induced emission enhancement in naphthalimides with special impact of the formyl group

**Fig. 3:**
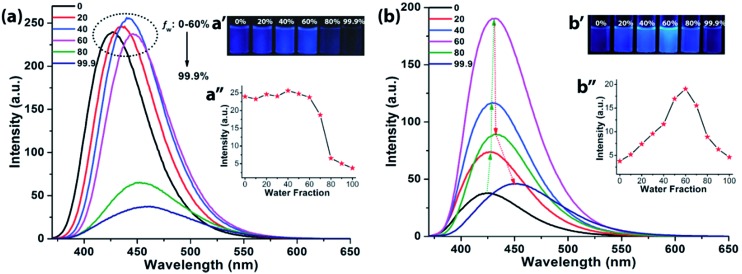
Fluorescence spectra of (a) **NIB** and (b) **NIBD** at different water fractions in DMF. Insets: digital photographs of (a′) **NIB** and (b′) **NIBD** at different water fractions in DMF at 365 nm irradiation along with plots of *λ*_emi,max_ (a′′) **NIB** and (b′′) **NIBD** at different water fractions in DMF (10 μM, excited at 355 nm). These experimental details validate the ACQ and AIEE characteristics of **NIB** and **NIBD** respectively.

**Fig. 4:**
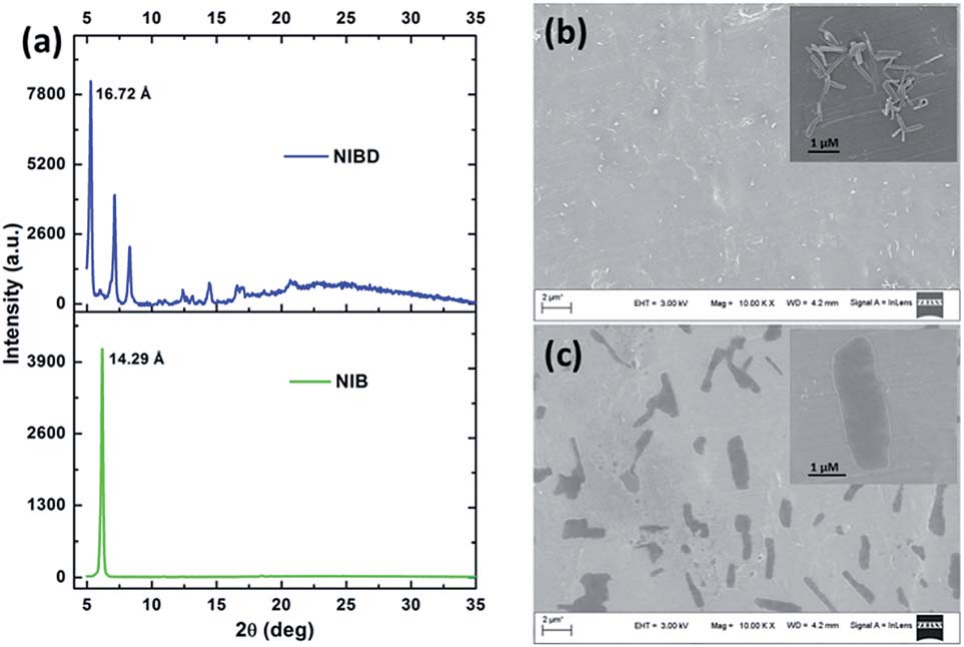
(a) X-ray diffractograms of thin films of **NIB** and **NIBD**. FE-SEM images of **NIBD** (b) and **NIB** (c) formed by the evaporation of the respective aggregates from a 99.9% water–0.1% DMF mixture on aluminium foil at room temperature (10 μM).

Although, **NIB** and **NIBD** were completely insoluble in water, the hexyl chain provides good solubility in most organic solvents. All the aggregation studies were performed in a DMF/water solvent system considering the good miscibility of DMF and water (Fig. 3). Both the naphthalimides displayed absorption and emission maxima at approximately 352 nm and 426 nm (*λ*_ex_ = 355 nm) in DMF respectively, which correspond to its molecular optical properties. Their fluorescence spectra were recorded by taking different *f*_w_ in DMF. The fluorescence intensity increased 4-fold on increasing the *f*_w_ up to 60% in **NIBD**, whereas the emission intensity remained constant in the case of **NIB**. At 99.8 *f*_w_, **NIB** became non-fluorescent, which clearly demonstrated the ACQ behavior of the naphthalimide (Fig. 3a). Although both the congeners showed similar types of absorption spectra, there was a huge diversity in their emission spectra (Fig. 3 and S1 of the ESI†). It is noteworthy that both the structurally similar naphthalimides have a phenyl ring and can undergo the RIM process. However, **NIB** possesses ACQ characteristics that could be due to the inability of the phenyl core to consume a significant fraction of the excitation energy in the solution state through dynamic rotation. In the case of **NIBD**, the formyl group could increase the relative mass of the phenyl moiety and at the same time it could undergo dynamic vibrations that may significantly induce non-radiative decay in the solution state. Besides, the presence/absence of the formyl group could affect their intermolecular interactions in the condensed state *via* H-bonding. To confirm the above supposition, wide-angle powder XRD patterns of the naphthalimides were recorded (Fig. 4a). **NIB** showed no significant peaks in the entire mid-angle region (except one peak at 2*θ* = 6.18°, corresponding to a *d*-spacing of 14.29 Å which is close to its molecular length); on the other hand several sharp peaks with comparable intensities were observed in **NIBD** owing to its highly crystalline nature.^7^ The images depicting the morphology of **NIB** and **NIBD** obtained from FESEM also supported the higher degree of regular arrangement in **NIBD** compared to **NIB** (Fig. 4b and c). **NIBD** formed a nanorod like morphology in water, whereas unsymmetrical micro-aggregates with variable sizes were obtained from **NIB**. The amorphous nature of **NIB** could be readily correlated with the absence of the formyl group, whereas in **NIBD**, the formyl moiety could strengthen the intermolecular hydrogen bonding, which may restrict the movement of aromatic parts more significantly; consequently, the energy loss through intramolecular motion can be suppressed more proficiently by the enhanced intermolecular interactions. These experimental analyses suggested that functional groups could also be manipulated to strengthen the RIM process and could serve as a new avenue to transform an ACQphore into an AIEEgen.

### Aqueous phase hydrazine detection at the parts per trillion level

**Fig. 5:**
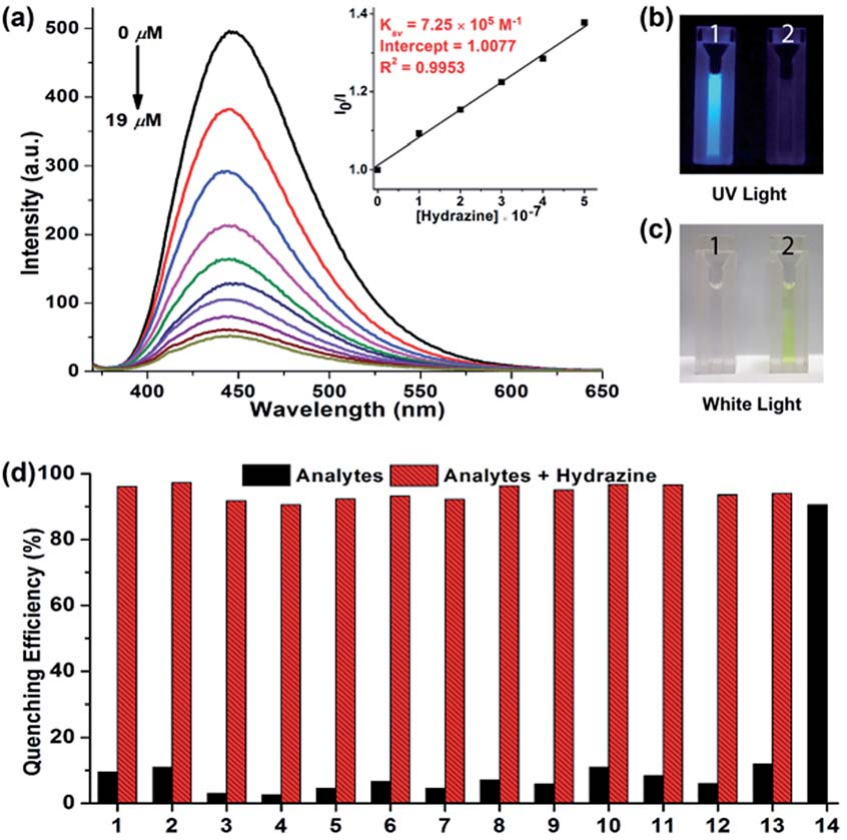
Aqueous phase detection of hydrazine. (a) Photo-luminescence spectra of **NIBD** (10 μM) with increasing concentration of hydrazine recorded after 15 min of incubation in PBS buffer at room temperature. Inset: Stern–Volmer plot of **NIBD** at different hydrazine concentrations. (b and c) Color change of **NIBD** nanoaggregates in PBS buffer before (1) and after (2) the addition of hydrazine under UV light (365 nm) and white light respectively. (d) Quenching efficiency of **NIBD** (10 μM) with several analytes (10 μM) in BPS buffer before and after the addition of hydrazine (10 μM): thiourea (1), urea (2), cysteine (3), homocystein (4), glutathione (5), leucine (6), glycine (7), dimethylamine (8), diethylamine (9), ethylenediamine (10), triethylamine (11), ammonium hydroxide (12), hydroxylamine (13), and hydrazine (14).

The fluorescent aggregates of **NIBD** (10 μM) formed in 10 mM PBS buffer (pH = 7.4) were considered for sensing studies. To investigate the optical response, fluorescence quenching titrations were carried out by subjecting different portions of hydrazine to **NIBD** (Fig. 5a). Each of the spectra was recorded after 15 min of incubation at room temperature. Almost 90% fluorescence quenching was observed upon adding just 9 μM solution of hydrazine. The disappearance of the blue luminescence of **NIBD** in the presence of hydrazine was clearly visible under UV light (Fig. 5b and c). The UV-visible spectrum of **NIBD** was recorded with and without hydrazine in aqueous media. The absorption band at 360 nm shifted towards the shorter wavelength region and a new band at 300 nm appeared corresponding to the hydrazone Schiff base derivative (see ESI, Fig. S2†). The transparent solution of **NIBD** transformed into yellow with the addition of hydrazine, which was visible to the naked eye (Fig. 5c). The quenching efficiency was obtained by fitting the Stern–Volmer equation linearly at lower concentration, and the Stern–Volmer constant (*K*_sv_) was found to be 7.25 × 10^5^ M^–1^ confirming the very high sensitivity of **NIBD** towards hydrazine (inset, Fig. 5a). The limit of detection (LOD) for hydrazine was calculated to be 2.54 × 10^–9^ M (81 ppt) using the standard method (*nσ*/*K*, *n* = 2 or 3, we have considered 3 here) which appeared to be the best value observed so far in the literature and much below the TLV (10 ppb) as identified by the US-EPA. (see ESI, Fig. S3 and Table S1†).1a,^8^

### Selectivity study for the detection of hydrazine in aqueous media

For achieving practicability of the probe along with sensitivity, the selectivity of **NIBD** towards hydrazine in an aquatic system was also tested. During this course, fluorescence titration experiments of **NIBD** with several amino acids (considering biological importance), amines, and anionic and cationic analytes were executed to monitor the selectivity of **NIBD** for hydrazine under identical conditions (Fig. 5d and Fig. S4 of the ESI†). It was observed that other amines, such as urea, thiourea and ethylenediamine, could not quench the fluorescence intensity of **NIBD** significantly (∼7–11%). Almost negligible quenching in fluorescence intensity of **NIBD** was observed with various anions (NO^3–^, NO^2–^, H_2_PO^4–^, H_3_PO^2–^, F^–^, Cl^–^, Br^–^, I^–^, OAc^–^, PPi^–^, CN^–^, and SCN^–^) and cations (Cs^+^, Mn^2+^, Co^2+^, Cr^2+^, Al^3+^, Cu^2+^, Cd^2+^, Pd^2+^, Zn^2+^, La^3+^, Fe^3+^, and Fe^2+^). Thus, the **NIBD** AIEEgen responded to hydrazine with outstanding sensitivity and selectivity even in the presence of commonly interfering analytes in pure aqueous medium which is a very unique feature of this probe.

### Elucidation of the detection mechanism *via* hydrazone Schiff base formation

**Fig. 6:**
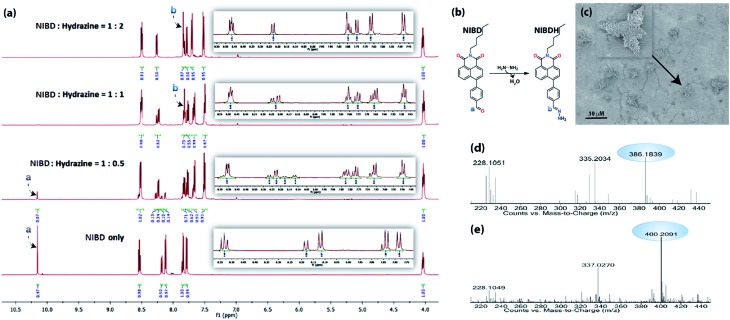
Elucidation of the sensing mechanism *via* formation of the hydrazone Schiff base complex. (a) ^1^H NMR spectra of **NIBD** (50 mM) recorded in the absence and presence of hydrazine at different concentrations (0.5, 1.0 and 2.0 equiv.) after 15 min of incubation in DMSO-*d*_6_. (b) Scheme representing the formation of the naphthalimide Schiff base derivative from **NIBD** at room temperature. (c) FESEM image of **NIBD** + hydrazine; the inset shows the corresponding magnified image. The FESEM images have been recorded by dropcasting the corresponding probe and analyte solution at 10 μM concentration from water on aluminium foil at room temperature. (d) ESI-HRMS of **NIBD**. (e) ESI-HRMS of **NIBDH**.

**Fig. 7:**
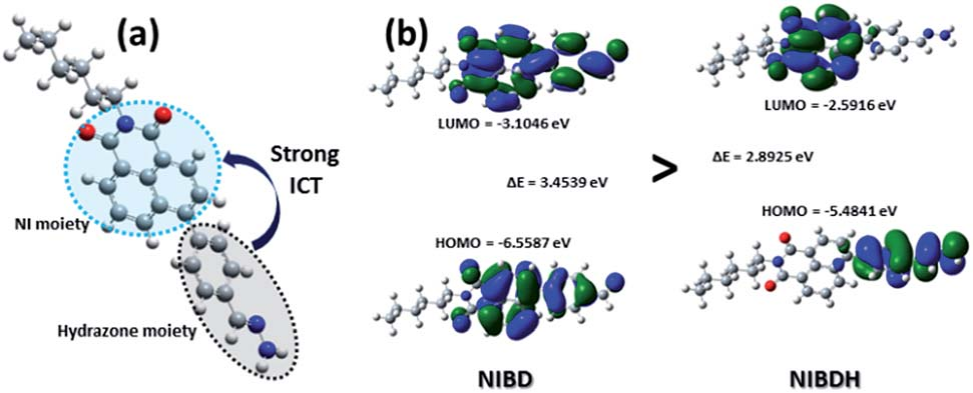
(a) Optimized structure of **NIBDH** and the co-regulation of response emission by the ICT mechanism. (b) Frontier molecular orbital energy of **NIBD** and **NIBDH** in the excited state. Computations were executed using time dependent density functional theory with the B3LYP exchange functional employing 6-31G* basis sets in the Gaussian 09 program.

Several experimental analyses were performed to understand the mode of interaction between **NIBD** and hydrazine. ^1^H NMR spectra of **NIBD** were recorded in DMSO-*d*_6_ at different hydrazine concentrations (0, 0.5, 1.0, and 2.0 equiv.) after 15 min of incubation (Fig. 6a and b). The addition of hydrazine causes a shift of the =CH (a) peak of the formyl group from *δ* = 10.15 ppm (^1^H) to *δ* = 7.80 ppm, which matched well with the =CH (b) peak of the hydrazone Schiff base derivative (**NIBDH**). Besides, the chemical shifts of the phenyl protons towards the more shielded region along with the total integration value supported the complete formation of the hydrazone Schiff base derivative. ESI-HRMS analysis of this hydrazone Schiff base derivative also revealed the formation of **NIBDH** with nearly 100% conversion and confirms the proposed mechanism (Fig. 6d and e). Furthermore, DLS studies performed in aqueous media showed an increase in the particle size after the formation of the Schiff base derivative (**NIBD**, *Z*_ave_ = 367.8 nm; **NIBDH**, *Z*_ave_ = 550.2 nm) and was confirmed from FESEM images (see ESI, Fig. S5 and S6†). Along with the size, a huge morphological transformation from nanorods to hastate shape microstructures (>10 μM) was witnessed after formation of **NIBDH** (Fig. 4b and 6c). To better understand the spectral changes of **NIBD** responding to hydrazine, theoretical calculations were performed using time dependent density functional theory (TDDFT) with the B3LYP(d) exchange functional, employing 6-31G* basis sets in a suite of the Gaussian 09 program (Fig. 7a and b).^9^ Both **NIBD** and its hydrazone Schiff base adduct (**NIBDH**) showed highly distinct structural conformation and electronic distribution in their excited state (Fig. 7b). In **NIBD**, electron densities over the HOMO and LUMO are dispersed all over the conjugated units without any significant charge separation. However, the π-electrons over the HOMO and LUMO are localized in the respective hydrazone and naphthalimide units in **NIBDH** with complete charge separation, suggesting that **NIBDH** is a typical ICT based fluorophore having strong donor (D) and accepter (A) units with push–pull interactions (Fig. 7).^9^ The hydrazone product **NIBDH** from the spontaneous reaction between **NIBD** and hydrazine retained a smaller energy difference between the LUMO and the HOMO (2.89 eV) than that of **NIBD** (3.45 eV) (Fig. 7b), which can be ascribed to the high feasibility of the ICT process from the electron-donating hydrazone group (D) to the electron-withdrawing naphthalimide group (A). Additionally, the huge increase in dipole moment (6.91 to 34.29) along with dihedral angle (43.31° to 90.78° angle between D and A units) after formation of the hydrazone Schiff base adduct also strongly supports the twisted ICT based fluorescence quenching in **NIBDH**.^9^ The strong donating nature of the hydrazone adduct is due to the conjugated –NH_2_ group with the aromatic ring. This was confirmed by the FTIR analysis of **NIBDH**, which showed a characteristic intense broad band at 3480 cm^–1^ assigned to the secondary amine (Fig. 1 and S7†).6*d*

### Detection of hydrazine vapour using **NIBD** loaded simple and cost-effective paper strips

**Fig. 8:**
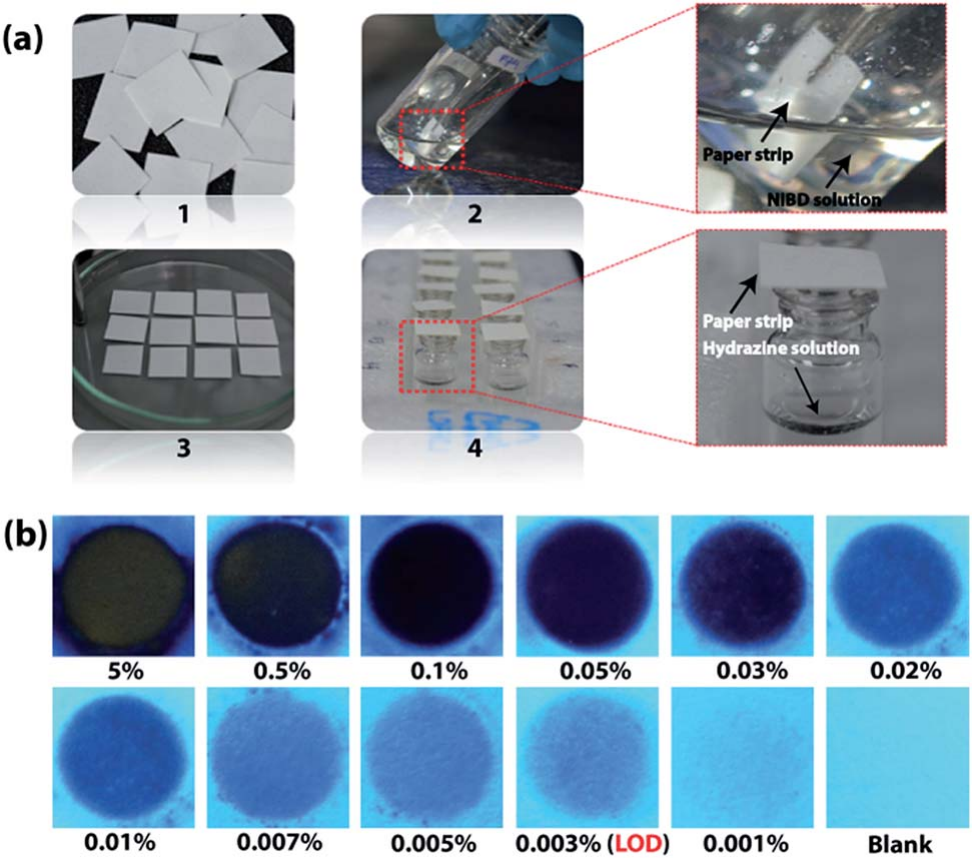
Fabrication of cost-effective paper strips for vapour phase on-site detection of hydrazine. (a) Representation of test strip fabrication for simple and cost-effective vapor phase detection of hydrazine using Whatman filter paper. (1) Whatman paper was cut into 1 cm × 1 cm pieces. (2) Dip-coating of Whatman paper strips in DMF solution of **NIBD** (20 mM) at room temperature for 10 seconds. (3) Drying of the paper strips on a hot-plate at 50 °C. (4) The mouth of the vials containing hydrazine hydrate solution of different concentrations was covered with the dried probe-loaded paper strips at room temperature. (b) Fluorescence color change of the probe-loaded test strips after exposing them to different hydrazine concentrations for 15 min. The fluorescence color variations were perceived by UV light illumination at 365 nm.

After gaining an unprecedented sensitivity by the fluorometric method, portable and simple paper strip based devices coated with the AIEEgen **NIBD** were fabricated to make these outcomes more practical for cost-effective on-site application. All of the fabrication steps are pictured in Fig. 8a. Whatman filter paper was cut into pieces of 1 cm × 1 cm dimensions and dipped in 20 mM DMF solution of **NIBD**. The paper strips were then dried on a hot plate at 50 °C for rapid evaporation of solvent before exposing them to hydrazine vapor. As shown in Fig. 8a(4), vials containing various concentrations of hydrazine hydrate solution (5% to 0.001%) were prepared and their mouths were covered with the dried probe-loaded paper strips for 15 min. A solvent blank was also taken as a control. Although hydrazine is highly volatile in nature, in previous reports, sophisticated special instruments that could work at low pressure for the effective vaporization of hydrazine have been used.6*d* This clearly depicts the low sensitivity and challenges faced by the existing probes. All the above hurdles have been overcome completely owing to the very high sensitivity of the newly designed AIEEgen. The filter papers were then pictured under 365 nm UV light irradiation. Different strengths of dark spots were witnessed on these test strips, which vary with the concentration of hydrazine present inside the vials (Fig. 8b).

The lowest amount of hydrazine vapor detectable (LOD) under UV-light irradiation was 0.003%, comprising the best result among the reported values (see ESI, Table S1†). In addition, vapor phase selectivity studies were also carried out with other competing vapor analytes such as ammonia, H_2_O_2_, triethylamine, butylamine, HCl and diethylamine, which confirmed the high selectivity of **NIBD** towards hydrazine (see ESI, Fig. S8†). These observations with extraordinary efficacy of the paper test strips endorse huge application potential for on-site, simple and cost-effective detection of hydrazine vapor.

### Application of the **NIBD** AIEEgen in two mammalian living cells to demonstrate its *in vitro* applicability and selectivity toward hydrazine

**Fig. 9:**
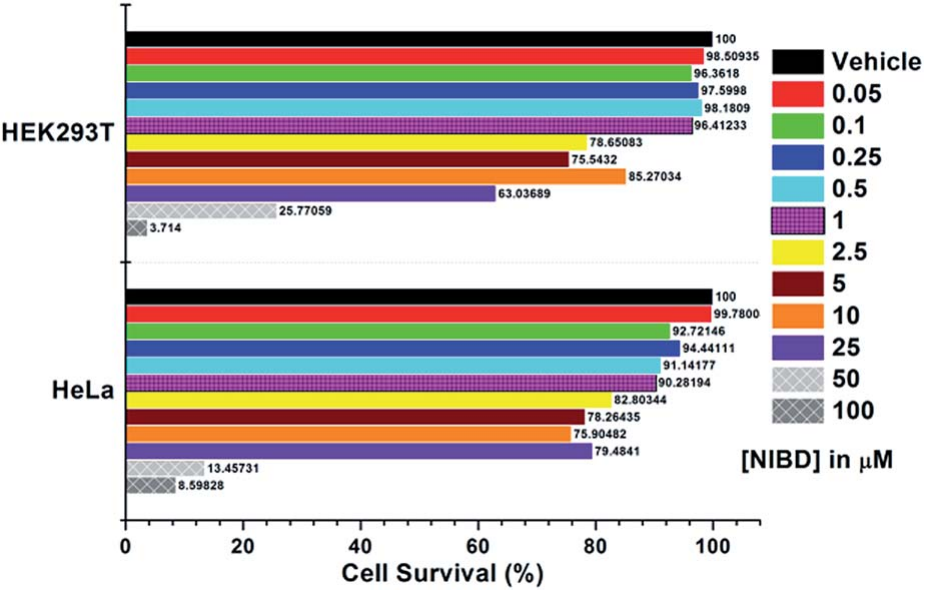
Cell cytotoxicity experiment for **NIBD***via* MTT assay. Both HEK293T and HeLa cells were plated in a 96-well polystyrene culture plate and allowed to grow for 24 h in DMEM supplemented with 10% fetal bovine serum (FBS), penicillin (1 unit per mL), and streptomycin (1 μg mL^–1^). The cells were maintained in a humidified atmosphere at 37 °C under 5% CO_2_ flow in an incubator. After 24 h, the existing medium was replaced with fresh medium containing different **NIBD** concentrations (0–100 μM) and incubated for another 24 h. Following 24 h of incubation, the medium was treated with 10 μL of methylthiazolyldiphenyl-tetrazolium bromide (MTT) solution (5 mg mL^–1^ in PBS) and kept as such for 4 h. The MTT-formazan crystals were then solubilized in DMSO and absorbance at 570 nm was recorded. Each of the data points is the average of three individual readings.

**Fig. 10:**
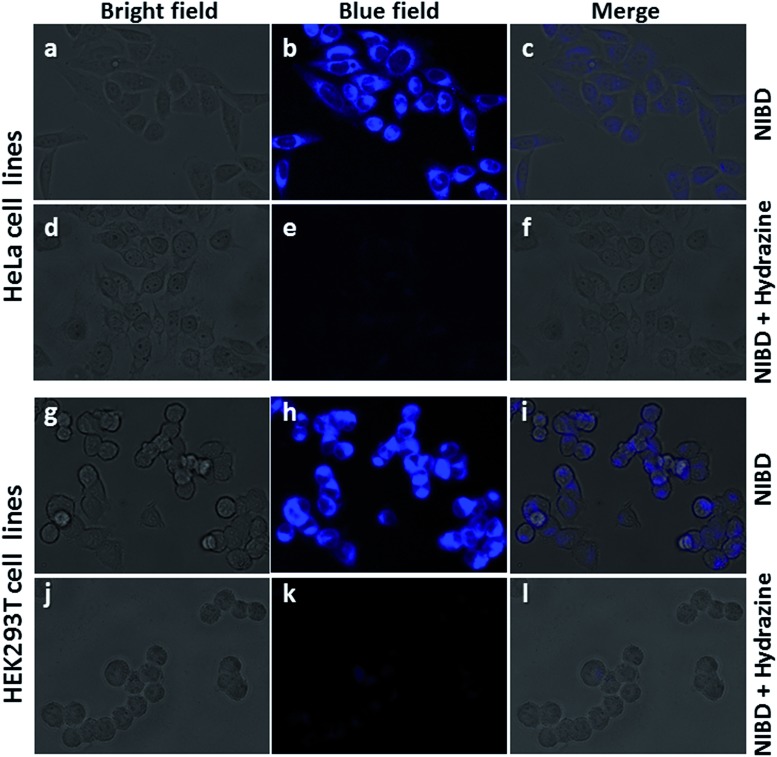
Fluorescence microscopy images of **NIBD** in response to hydrazine addition in two mammalian cell lines (HeLa and HEK293T). Both the cells were treated with 1 μM **NIBD** in a medium (DMEM supplemented with 10% fetal bovine serum (FBS), penicillin (1 unit per mL), and streptomycin (1 μg mL^–1^)) and were incubated in a humidified atmosphere at 37 °C under 5% CO_2_ flow for 90 min, exchanged into fresh medium containing hydrazine and incubated for another 2 h. Bright field, blue and merged images of HeLa (a–c) with **NIBD** and (d–f) **NIBD** + hydrazine respectively. Bright field, blue and merged images of HEK293T (g–i) with **NIBD** and (j–l) **NIBD** + hydrazine respectively.

The applicability of this highly selective and novel naphthalimide AIEEgen (**NIBD**) was also examined in living cells for the detection of hydrazine. In this instant, two mammalian cell lines, *i.e.* HeLa (human cervical cancer cell line), and HEK293T (Human embryonic kidney cell line), procured from the repository of the National Centre for Cell Science (NCCS) Pune, India were employed to study the *in vitro* selectivity and potential of **NIBD** toward hydrazine. Before the hydrazine detection study, the effect of **NIBD** towards the cell lines was determined by treating both the cells with different concentrations (from 0.05 μM to 100 μM) of the same AIEEgen. MTT assay was performed to check the cytotoxicity of **NIBD** (Fig. 9) and simultaneously fluorescence imaging was carried out for each of the concentrations. Considering these results, a lower but effective concentration (1 μM) was selected for further imaging and hydrazine detection study. Both the cell lines were treated independently with **NIBD** (1 μM) for 90 minutes and then hydrazine was subjected to the media. Images were acquired using a fluorescence microscope after 2 h of post-incubation. As revealed in Fig. 10, the strong blue fluorescence of **NIBD** incubated cells disappeared almost completely in the presence of hydrazine after 2 h due to the formation of the non-fluorescent hydrazone Schiff base derivative (**NIBDH**). This result lends further support to the high selectivity of the hydrazine induced *in vitro* complexation of **NIBD** to form **NIBDH** and shows no considerable interference from other biologically abundant metal ions and amino acids present in the medium.

It is also worth mentioning that **NIBD** could image the cells with high fluorescence in a wash-free medium owing to the application of a very low concentration of the AIEEgenic probe (see ESI, Fig. S9†). These results led us to believe that almost all the probes were engulfed by the cells actively within 60 min of incubation, thereby resulting in almost no background fluorescence in a wash-free medium. These results also confirm that the **NIBD** AIEEgen could efficiently detect and bind hydrazine *in vitro* to form hydrazone Schiff base derivative **NIBDH**, a non-fluorescent complex even in a physiological environment, thereby demonstrating the unique sensitivity and selectivity in multiple cell lines as well as providing avenues for cellular imaging, sensing, monitoring and clinical diagnosis.

## Conclusions

In conclusion, two core substituted naphthalimide congeners have been synthesized, providing a new insight into an exceptional ACQ to AIEE transformation. Conclusive analyses suggested that specific functional groups (aldehyde in this case) could be precisely manipulated to reinforce the RIM process in ACQphore systems and could efficiently transform them into AIEEgenic systems, a concept unexplored previously.

Although **NIB** possessed a phenyl ring conjugated with a C–C single bond that could rotate dynamically, similar to **NIBD**, it is unable to consume the major fraction of the excitation energy. However, the presence of a formyl group in **NIBD** increases the relative mass of the pendent phenyl ring and could consume a greater fraction of the excitation energy in the solution state *via* dynamic vibration. Besides, the formyl group could strengthen the intermolecular interactions in the aggregated state *via* intermolecular hydrogen bonding which makes **NIBD** an AIEE-active molecule.

Additionally, the newly designed naphthalimide AIEEgen with the formyl group selectively detects hydrazine at the parts per trillion level (81 ppt) in pure aqueous media. The probe could spontaneously react with hydrazine to form a hydrazone Schiff base derivative resulting in fluorescence quenching at room temperature. An extremely high detection limit was also accomplished in the vapor phase (0.003%) by fabricating Whatman paper strips which could provide a simple, portable and cost-effective method for on-site detection of hydrazine. The *in vitro* efficacy of the AIEEgenic probe towards hydrazine was also demonstrated in two mammalian cell lines (HeLa and HEK293T) with a high signal-to-noise response. Along with good imaging capability, the probe responds to hydrazine without any interference from biologically abundant metal ions and amino acids.

Further work is underway to explore the impact of other functional groups on the condensed state emission properties of naphthalimides and other ACQ systems. We are also trying to expand the scope of this Schiff base complexation for developing probes for imaging of hydrazine metabolism and therapeutic applications. This complexation could be potentially applicable to the hydrazine scaffold and can be envisioned for a broader utility of this chemistry in elucidating new foundations and targets of hydrazine metabolism in biological pathways.

## Conflicts of interest

There are no conflicts to declare.

## Supplementary Material

Supplementary information
