# Supplementary material for: Aldehyde group driven aggregation-induced enhanced emission in naphthalimides and its application for ultradetection of hydrazine on multiple platforms
Source: Chem Sci. 2018 Apr 6;9(16):3978–85. doi: 10.1039/c8sc00643a (PMC5944821; doi:10.1039/c8sc00643a)
Supplement: Supplementary file 1 [file SC-009-C8SC00643A-s001.pdf]

Electronic Supplementary Material (ESI) for Chemical Science.
This journal is © The Royal Society of Chemistry 2018

Electronic Supplementary Information

Aldehyde Group Driven Aggregation-Induced Enhanced Emission in Naphthalimides and its Application for Ultradetection of Hydrazine on Multiple Platforms

Niranjan Meher,[a] Swagatika Panda,[b] Sachin Kumar,[b] and Parameswar Krishnan Iyer*,[a,c]

[a]*Department of Chemistry, Indian Institute of Technology Guwahati, Guwahati-781039. Assam. India*
[b]*Department of Bioscience and Bioengineering, Indian Institute of Technology Guwahati, Guwahati-781039. Assam. India*
[c]*Center for Nanotechnology, Indian Institute of Technology Guwahati, Guwahati-781039, Assam, India*

AUTHOR INFORMATION (*Corresponding Author*)

**E-mail: pki@iitg.ernet.in; Fax: +91 361 258 2349 (P. K. Iyer).*

Sl. No.	Contents	Page No.
1	Experimental Section	S2
2	Comparison table on hydrazine detection	S6
3	UV-Visible spectra	S7
4	Sensing studies	S8
5	DLS Studies	S10
6	IR spectra	S11
7	Selectivity in vapor phase	S11
8	Cell imaging in wash-free medium	S12
9	^{1}H-NMR, ^{13}C-NMR and Mass Spectra	S12
10	References	S18

1. Experimental Section

1.1. Materials and methods

All starting materials and reagents (viz: 4-bromo-1,8-naphthalene anhydride, 4-formylphenylboronic acid, metal salt, amines, amino acids) were purchased from Sigma Aldrich (INDIA) and were of reagent grade. HPLC grade solvents were purchased from Zenith India and Northeast Chemicals. NMR (^{1}H, ^{13}C) spectra were recorded with a Varian-AS400 NMR spectrometer or Bruker Avance 600 MHz spectrometer. All solutions for ^{1}H and ^{13}C spectra were obtained taking residual solvent signal as internal reference. Electro spray ionization mass (ESI-MS) spectra were recorded on a Waters (Micro mass MS-Technologies) Q-Tof MS Analyzer spectrometer. Microbalance (± 0.1mg) and volumetric glassware were used for the preparation of solutions. UV/vis and PL spectra were recorded on a Perkin-Elmer Model Lambda-750 spectrophotometer and a Horiba Fluoromax-4 spectrofluorometer respectively using 4 mm quartz cuvettes at 298 K. Malvern Zetasizer instrument was used to measure the hydrodynamic diameter of the compounds. Life-time measurements were performed using a MicroTime-200 instrument. FE-SEM samples were made using drop-cast method from Water and were left for drying at room temperature. FE-SEM-images were obtained on Sigma Carl ZEISS field emission scanning electron microscope.

1.2. Cytotoxicity Assay

Cytotoxicity Assay of **NIBD** was performed using two different cell lines (HeLa and HEK293T), procured from the repository of National Centre for Cell Science (NCCS) Pune, India. Both HeLa and HEK293T cell were seeded into two different 96-well plates separately at an average of 15,000 and 12,000 cells/well in 150 μL medium, respectively. The cells were allowed to grow for 24 h at

37 °C under 5% CO_2 flow. Further, each of the well containing cells was treated with **NIBD** in a concentration range 0-100 µM in 150 µL media. After 24 h of incubation, 10 µL of Methylthiazolyldiphenyl-tetrazolium bromide (MTT) solution (5 mg/mL in PBS) was added to each well. The MTT treated cells were further incubated for 4 h at 37 °C under 5% CO_2 flow. Then, the medium containing unreacted MTT was removed and 100 µL of DMSO was added to each of the well to solubilize the formazan crystal formed from MTT by mitochondrial reductase enzyme of actively metabolizing cells. The absorbance was recorded at 570 nm in Microplate Spectrophotometer (Thermo Scientific™, USA) after incubation for 30 min at 37 °C shaker incubator.

1.3. Cell culture and imaging

Both HeLa and HEK293T cell were seeded in a 24-well cell cultured dish for 18 h in Dulbecco's modified Eagle's medium (DMEM) supplemented with 10% fetal bovine serum (FBS), penicillin (1 units/mL), and streptomycin (1 µg/mL) maintained at humidified atmosphere of 37 ^{o}C under 5% CO_2 flow in an incubator. The cells were treated and incubated with 1 µM of **NIBD** at 37 °C under 5% CO_2 for 2 h. To remove remaining extracellular dye, the cells were washed two times with PBS and then cell images were recorded before and after the treatment of of hydrazine directly into the medium using a fluorescence microscope from ThermoFisher Scientific, USA. Blue emission was collected with a 446 nm window.

1.4. Syntheses of Compounds

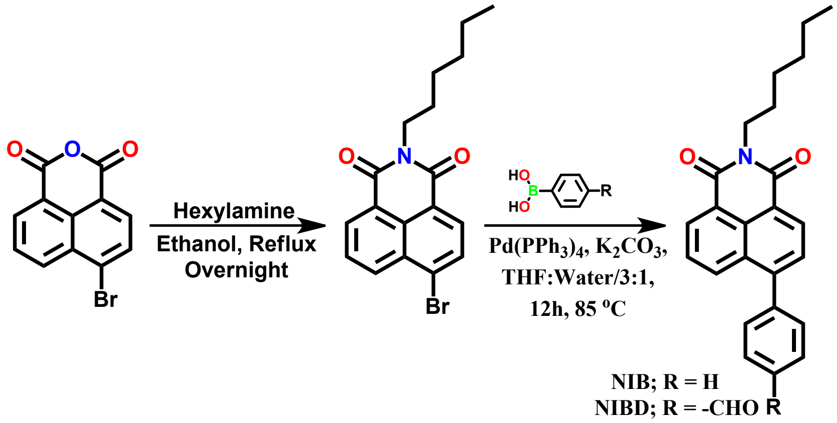

Scheme S1: Synthetic root to **NIB and NIBD**.

Compound 6N: 4-bromo-1,8-naphthalic anhydride (554.2 mg, 2 m.mol) was taken in ethanol (20 mL) and hexylamine (202.38 mg, 2 m.mol) was added to it at room temperature. The suspension was heated at 85 °C with vigorous stirring for 8h. Then the mixture was cooled to room temperature and evaporated under reduced pressure. The mixture was extracted with chloroform and washed with water. Then it was dried over anhydrous Na_2SO_4 and concentrated. After that it was finally purified by column over silica gel with 2% ethyl acetate in hexane as eluent which gives pure **6N.**

Greenish yellow solid (630 mg, 87% yield). ^{1}H NMR (400 MHz, $CDCl_3$, δ ppm) 0.87 (t, 3H), 1.31 (m, 6H), 1.70 (m, 2H), 4.12 (t, 3H) 7.77 (t, 1H), 7.94 (d, 1H), 8.32 (d, 1H), 8.46 (d, 1H), 8.57 (d, 1H). ^{13}C NMR (100.00 MHz, CDCl3, δ ppm) 14.25, 22.74, 26.96, 28.18, 31.71, 40.78, 122.34, 123.21, 128.15, 128.98, 130.24, 130.61, 131.16, 131.25, 132.06, 133.22, 163.60, 163.63. HRMS (+ESI): Calculated for $C_{18}H_{18}BrNO_2$ 359.0521 $[M]^+$, Found 360.0600 $[M+1]^+$.

Compound NIBD and NIB: Compound **6N** (0.180 g, 0.5 m.mol) and 4-formylphenylboronic acid (0.150 g, 1 m.mol for **NIBD**) or phenylboronic acid (0.122 g, 1 m.mol for **NIB**) were dissolved into 6 mL of THF in 50 ml independent round bottom flask, and then 2 mL of 2.0 M potassium carbonate solution was added to each of the flask. The mixture solutions was degassed with nitrogen for 15 min and then 5 mg of catalyst $Pd(PPh_3)_4$ was added to them. The mixtures were stirred at 85 °C under nitrogen atmosphere and the progress of reactions was monitored by taking TLC. After 15 h, the solutions were cooled and extracted with $CHCl_3$ after adding 50 mL of water. The organic layers were dried over anhydrous sodium sulfate. The solvent was removed and were purified by column chromatography, yielding a light greenish solid for **NIBD** (yield 0.150 g, 78%) and a yellow-green solid for **NIB** (yield 0.127 g, 71%).

NIBD: ^{1}H NMR (600 MHz, DMSO, δ ppm) 0.85 (t, 3H), 1.29 (m, 6H), 1.62 (m, 2H), 4.03 (t, 2H), 7.78 (d, 2H), 7.83 (m, 2H), 8.11 (dd, 2H), 8.13 (d, 1H), 8.53 (m, 2H), 10.15 (s, 1H). ^{13}C NMR (100.00 MHz, $CDCl_3$, δ ppm) 14.10, 22.68, 26.91, 28.17, 31.66, 40.69, 122.71, 123.19, 127.37, 127.92, 128.71, 129.77, 129.97, 130.16, 130.60, 130.72, 130.86, 136.22, 145.04, 145.17, 163.97, 164.18, 191.75. Calculated for $C_{25}H_{23}NO_3$ 385.1678 $[M]^+$, Found 386.1839 $[M+H]^+$.

NIB: ^{1}H NMR (600 MHz, $CDCl_3$, δ ppm) 0.90 (t, 3H), 1.34 (m, 4H), 1.45 (m, 2H), 1.75 (m, 2H), 4.20 (t, 2H), 7.53 (m, 5H), 7.69 (m, 2H), 8.26 (d, 1H), 8.64 (m, 2H). ^{13}C NMR (150.00 MHz, $CDCl_3$, δ ppm) 14.22, 22.72, 26.96, 28.23, 31.72, 40.67, 121.94, 123.06, 126.95, 127.97, 128.59, 128.79, 130.01, 130.18, 130.93, 131.28, 132.73, 138.97, 146.97, 164.27, 164.47. Calculated for $C_{25}H_{23}NO_3$ 357.1858 $[M]^+$, Found 358.1806 $[M+H]^+$.

2. Comparison table on hydrazine detection

Table S1. A comparative study of the K_{sv} and the detection limit (both in solution and vapor phase) along with the material and medium used for hydrazine detection with the existing state of art.[1-17]

Publication	Material Used	K_{sv} (M^{-1})	Detection Limit (Liquid Phase)	Medium Used	Detection Limit (Vapor Phase)
**Present Work**	**Naphthalimide AIEEgens**	**7.25×10^5**	**2.54 nM (81 ppt)**	**PBS Buffer**	**0.003%**
J. Mater. Chem. C, 2016, 4, 2834	Tetraphenylethy lene moiety	--	143 ppb	DMSO–PBS buffer (9/1, v/v);	0.1%
Chem. Commun., 2016, 52, 6166	Pthalimide based probe	--	1.5 ppb	In HEPES buffer using Triton X 100 surfactant	--
Sensors and Actuators B, 2016, 232, 369–374	Coumarin/benzo pyrylium based probe	--	47 nM (1.5 ppb)	DMSO:PBS (2:3, v/v)	1%
RSC Adv., 2016, 6, 70855	Thiadiazol based moiety	--	84 nM (2.9 ppb)	DMSO–H2O (6/4, v/v)	--
Anal. Chem. 2015, 87, 9101–9107	Hemicyanine based probe	--	0.17 µM (5.4 ppb)	HEPES buffer-DMSO (8/2, v/v)	--
Org. Biomol. Chem., 2015, 13, 5344–5348	ESIPT-based fluorescent	--	0.147 µM	PBS buffer-ethanol (99/1, v/v)	--
Chem. Commun., 2014, 50, 1485	Naphthalimide moiety	--	8.8 nM (0.3 ppb)	DMSO-H_2O (6/4, v/v).	10%
J. Mater. Chem. B, 2014, 2, 7344	Dansyl-based	1.03×10^4	188 nM (6.01 ppb)	DMSO-HEPES buffer (9/1, v/v)	0.01%
Anal. Chem. 2014, 86, 4611−4617	Phthalimide based probe	--	0.1 µM (3.2 ppb)	H2O/DMSO (v/v, 3:7)	0.1%
J. Mater. Chem. B, 2014, 2, 1846	ICT-based probe	--	12 nM	DMF–Tris HCl buffer (7/3, v/v)	--
RSC Adv., 2014, 4, 41807–41811	Benzo[*d*] oxazole	--	84 nM (2.7 ppb)	CH3CN–HEPES buffer (1/2, v/v)	10%
Chem. Sci., 2013, 4, 4121-4126	Naphthalimide moiety	--	100 nM (3.2 ppb)	CH_3CN	Excess
Org. Lett., 2013, 15, 5412–5415	Benzothiazole based probe	--	66 nM (2.2 ppb)	CH_3CN:H_2O (2/3, v/v)	--
Org. Lett., 2013,	Cyanine dye	5.68×10^5	25 nM	DMSO–Acetate	--

15, 4022–4025	derivative		(0.81 ppb),	buffer (9/1, v/v)	
RSC Adv., 2013, 3, 18872–18877	Carbazole based probe	--	1.02 µM	CH3CN–H2O (8 : 2, v/v)	--
Org. Lett., 2011, 13, 5260–5263	Coumarin dye	--	2.46 µM (0.08 ppm)	DMSO–Acetate buffer (7/3, v/v)	--
Org. Biomol. Chem., 2013, 11, 2961	Dichlorofluorescein and resorufin acetates	--	90 nM (2.9 ppb)	DMSO-tris buffer (1 : 1, v/v)	--

3. UV-Visible spectrum

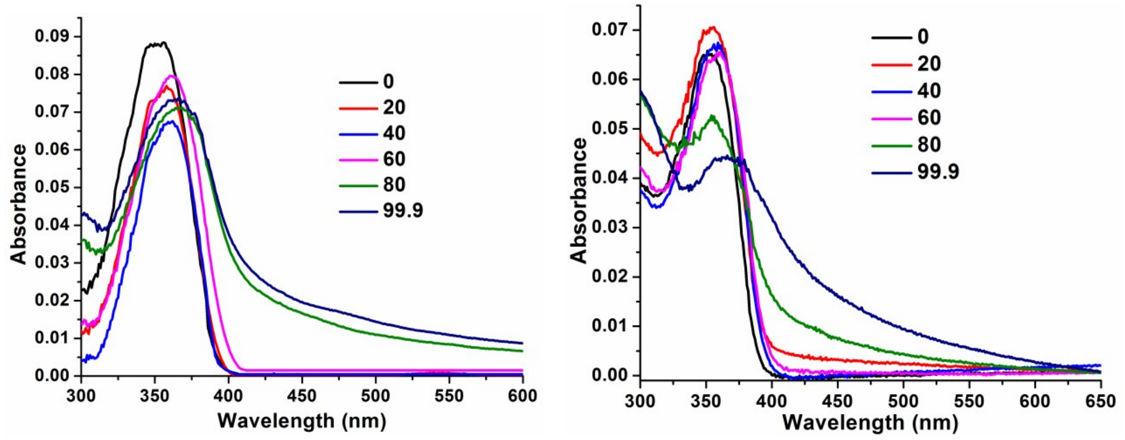

Figure S1. UV-Visible spectrum of (Left) **NIB** and (Right) **NIBD** taken at different water fractions in DMF (10 µM).

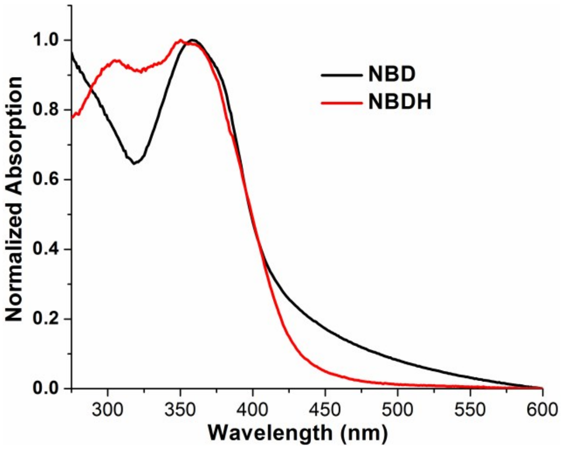

Figure S2. Normalized UV-Visible spectrum of **NIBD** and **NIBD**H in 99.9% aqueous media.

4. Sensing studies

The monomer **NIBD** (3.8 mg) stock solution was prepared at a concentration of 10 mM in 1 mL DMF. This was diluted to 10 µM for each titration in a 1 mL cuvette. The stock solutions of various analytes were (1 mM) were introduced in portions and the fluorescence intensity changes were recorded at pH 7.4 at room temperature (excitation wavelength: 355 nm) in PBS-buffer.

4.1. Calculation of Detection Limit

For calculating detection limit, different samples of **NIBD** (10 µM) each containing hydrazine (0 µM, 0.1 µM, 0.2 µM, 0.3 µM, 0.4 µM and 0.5 µM) were prepared separately and fluorescence spectrum was then recorded for each sample by exciting at 355 nm. The detection limit plot for hydrazine was obtained by plotting change in the fluorescence intensity versus concentration of hydrazine. The curve shows a linear relationship and the correlation coefficient (R^2) via linear regression analysis was calculated to be 0.9843. The limit of detection (LOD) was then calculated using the equation $3\sigma/K$, where σ represents the standard deviation in the intensity of **NIBD** in the absence of hydrazine and K symbolizes slope of the equation.

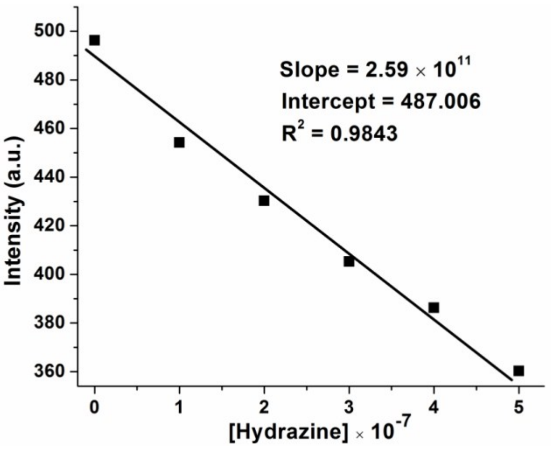

Figure S3. Fluorescence response of **NIBD** (10 μM) taken in aqueous buffer solution (pH 7.4) as a function of hydrazine concentration.

4.2. Selectivity study

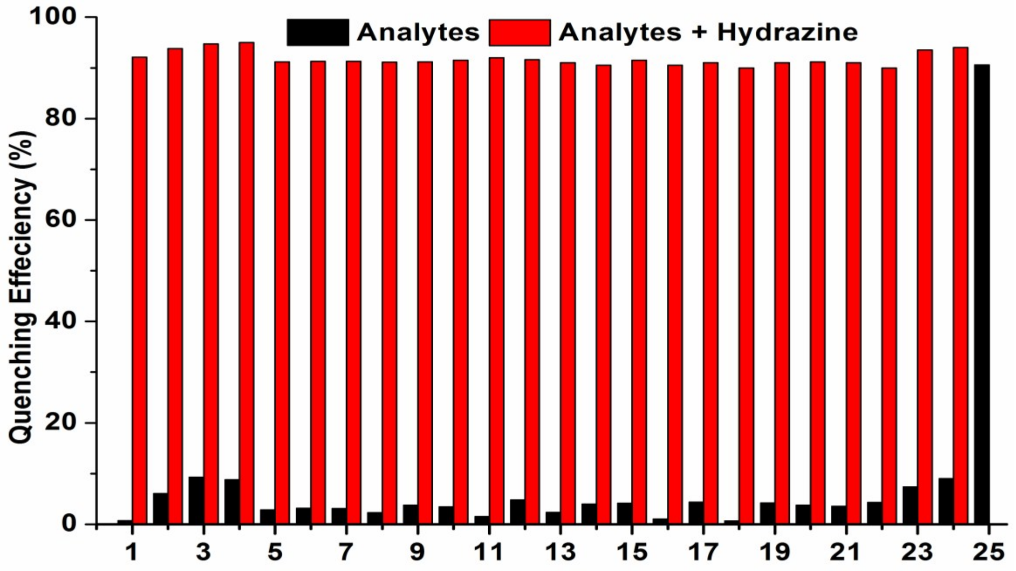

Figure S4. Quenching efficiency of **NIBD** (10 μM) with several anions and cations (10 μM) in BPS buffer before and after the addition of hydrazine (10 μM). NO_3^- (1), NO_2^- (2), $H_2PO_4^-$ (3), $H_3PO_2^-$ (4), F^- (5), Cl^- (6), Br^- (7), I^- (8), OAc^- (9), PPi (10), CN^- (11), SCN^- (12), Cs^+ (13), Mn^{2+}

(14), Co^{2+} (15), Cr^{2+} (16), Al^{3+} (17), Cu^{2+} (18), Cd^{2+} (19), Pd^{2+} (20), Zn^{2+} (21), La^{3+} (22), Fe^{3+} (23), Fe^{2+} (24), Hydrazine (25).

5. DLS Studies

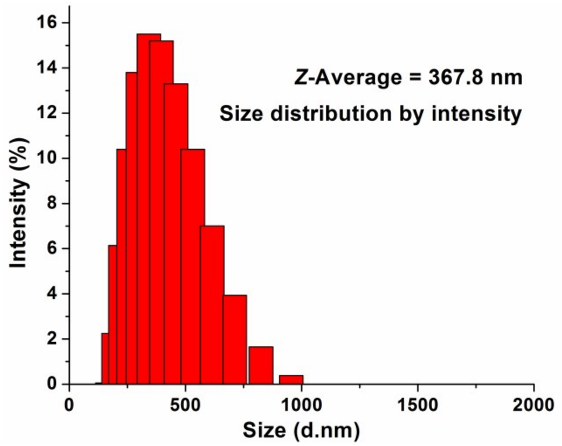

Figure S5. Size distribution by DLS of **NIBD** aggregate (10 μM) in water at 25 ^{o}C.

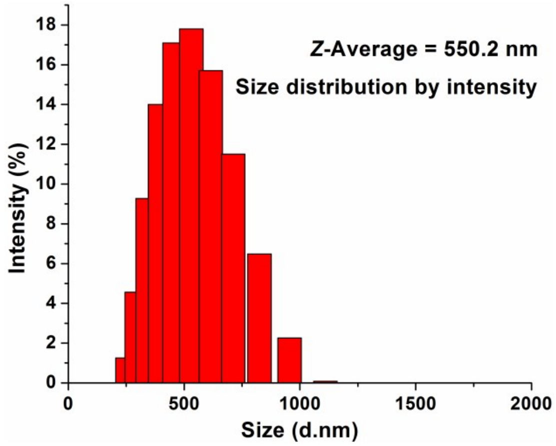

Figure S6. Size distribution by DLS of **NIBDH** aggregate (10 μM) in water at 25 ^{o}C.

6. IR spectra

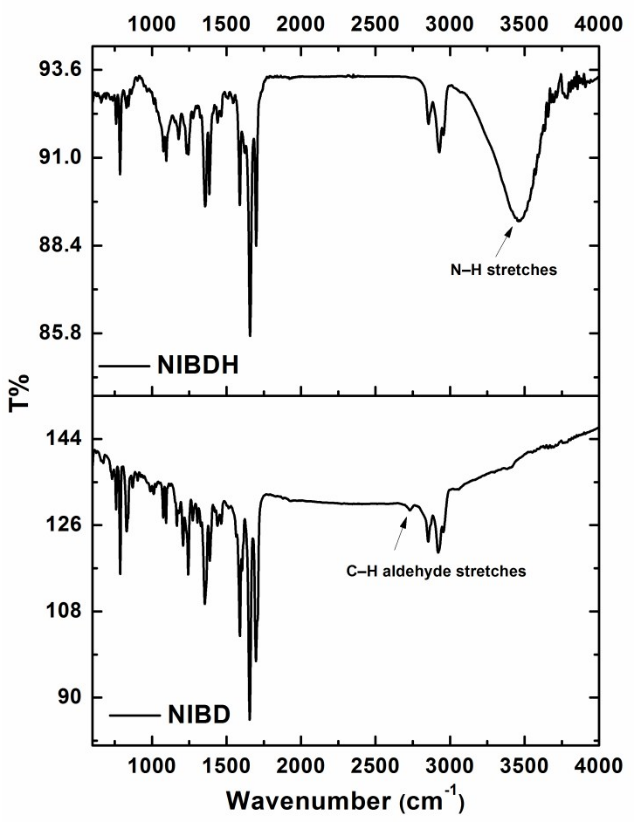

Figure S7. IR spectra of **NIBD** and **NIBD**H.

7. Selectivity in vapor phase

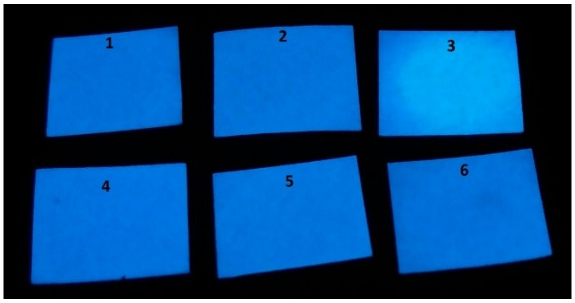

Figure S8. Digital photographs showing the fluorescence response of the **NIBD**-deep coated test strips after exposing to different vapor analytes for 15 min. (1) Diethylamine, (2) ammonia, (3) butylamine, (4) triethylamine, (5) HCl, (6) H_2O_2

8. Cell imaging in wash-free medium

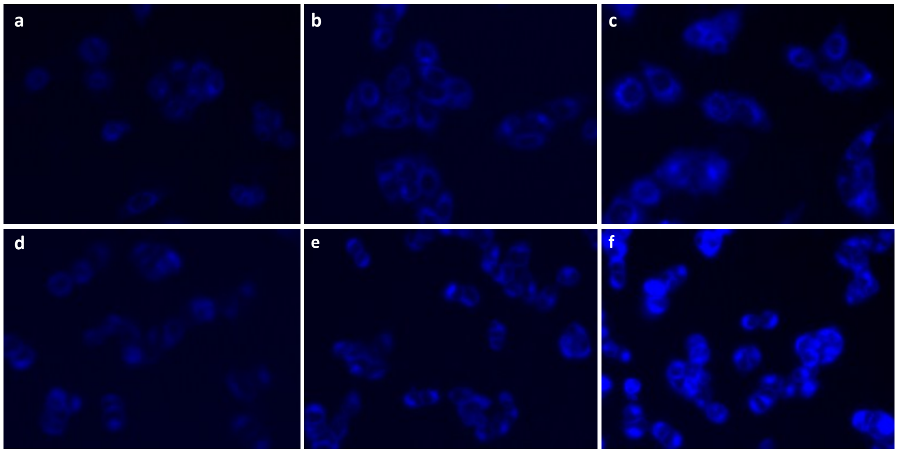

Figure S9. Fluorescence microscopy images of **NIBD** in a wash-free medium in HeLa and HEK293T cells at different concentrations. Fluorescence microscopy images of HeLa (a-c) and HEK293T (d-f) cells at 0.5, 1 and 2.5 µM respectively. Images were recorded after 90 min of incubation at 37 °C under 5% CO_2 flow.

9. ^{1}H-NMR, ^{13}C-NMR and Mass Spectra

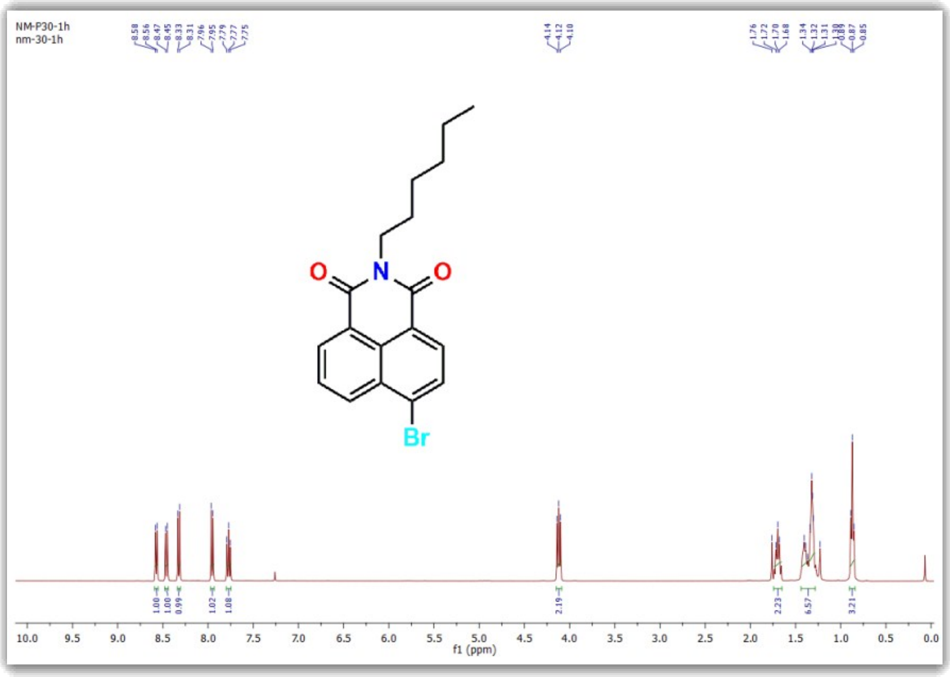

^{1}H NMR of 6N

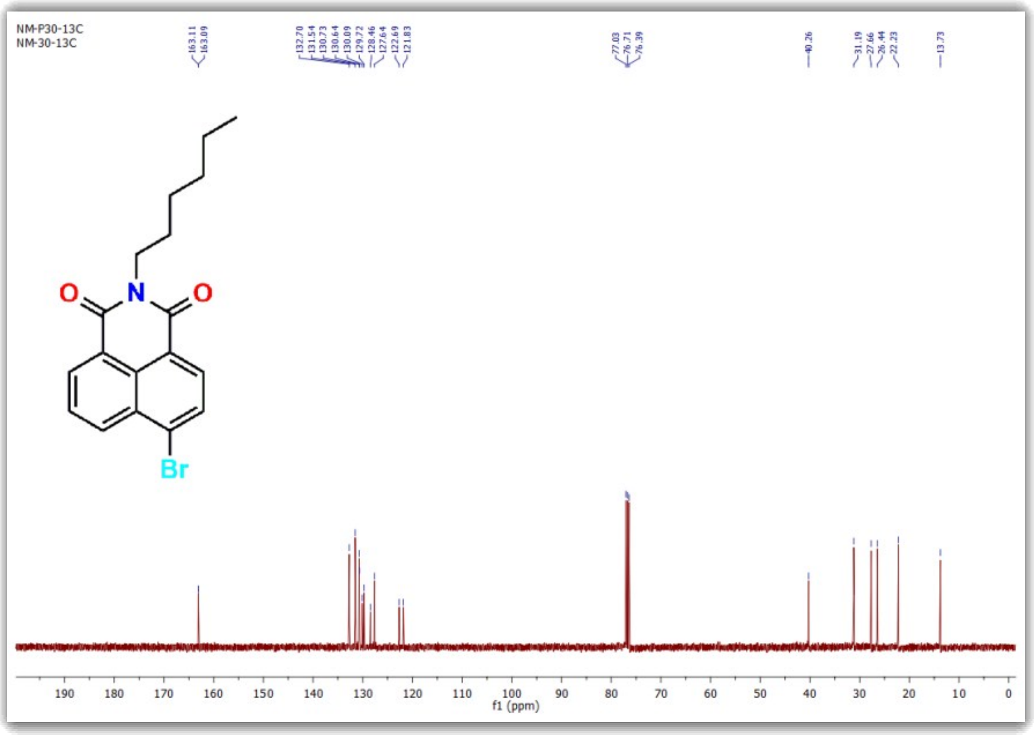

^{13}C NMR of 6N

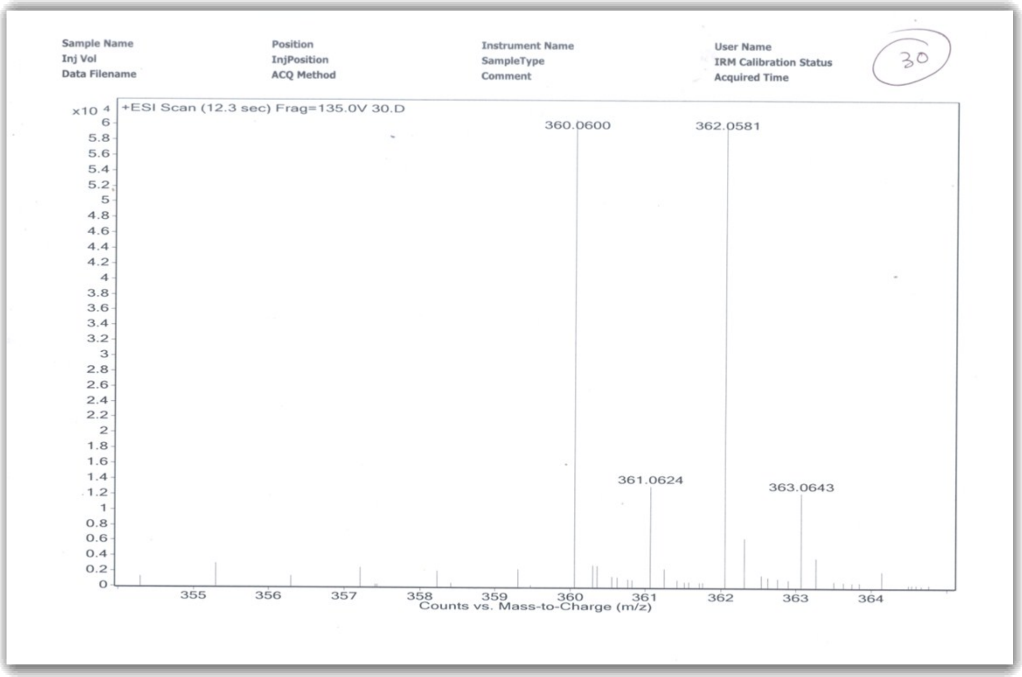

Mass Spectra of 6N

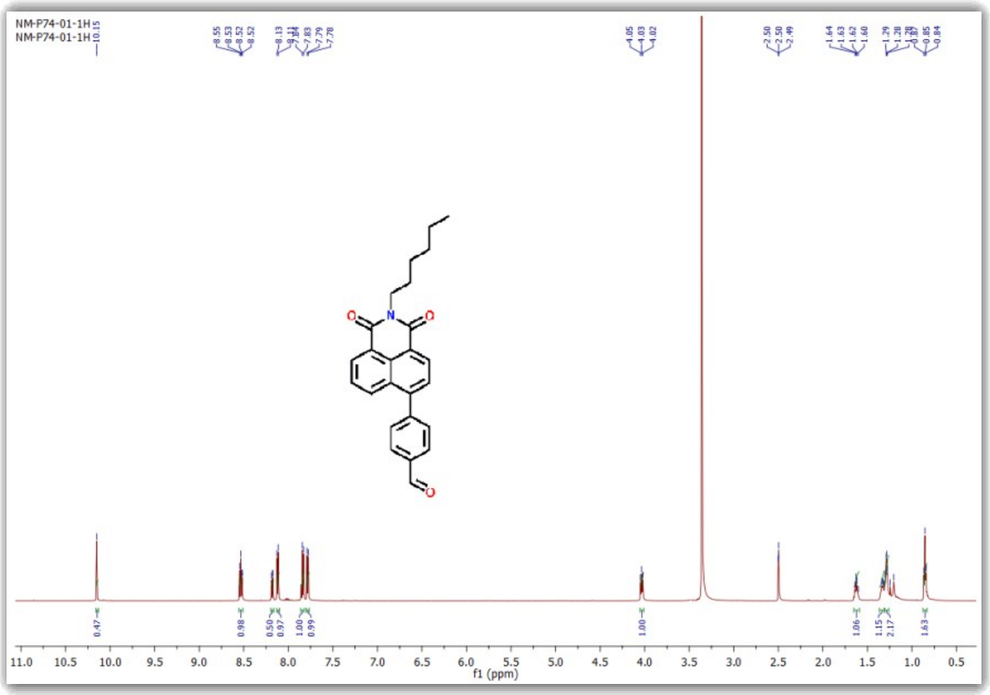

^{1}H NMR of NIBD

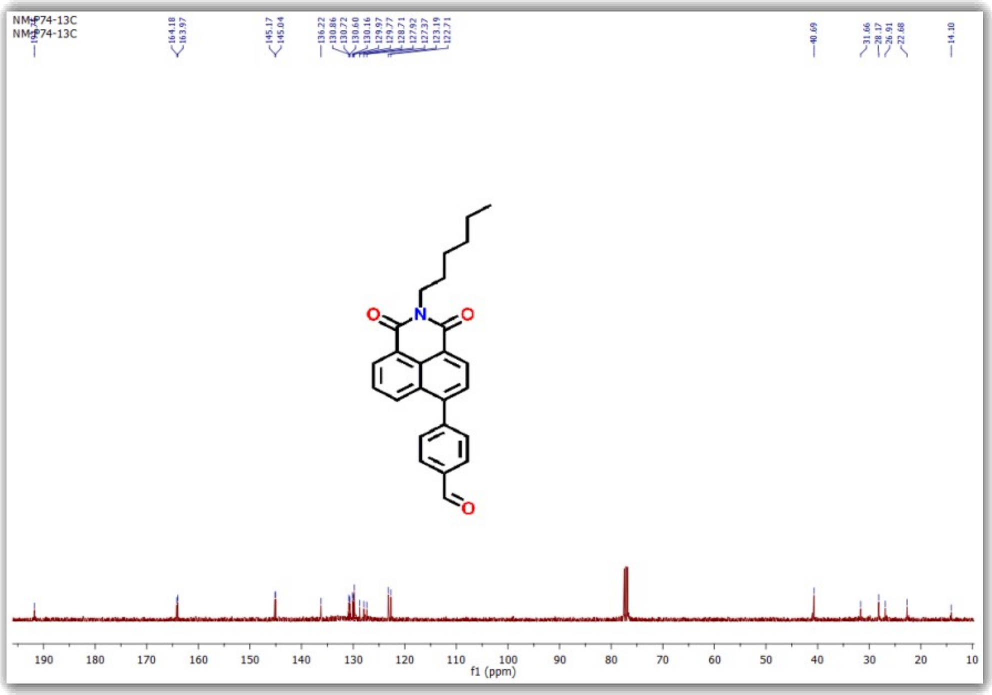

^{13}C NMR of NIBD

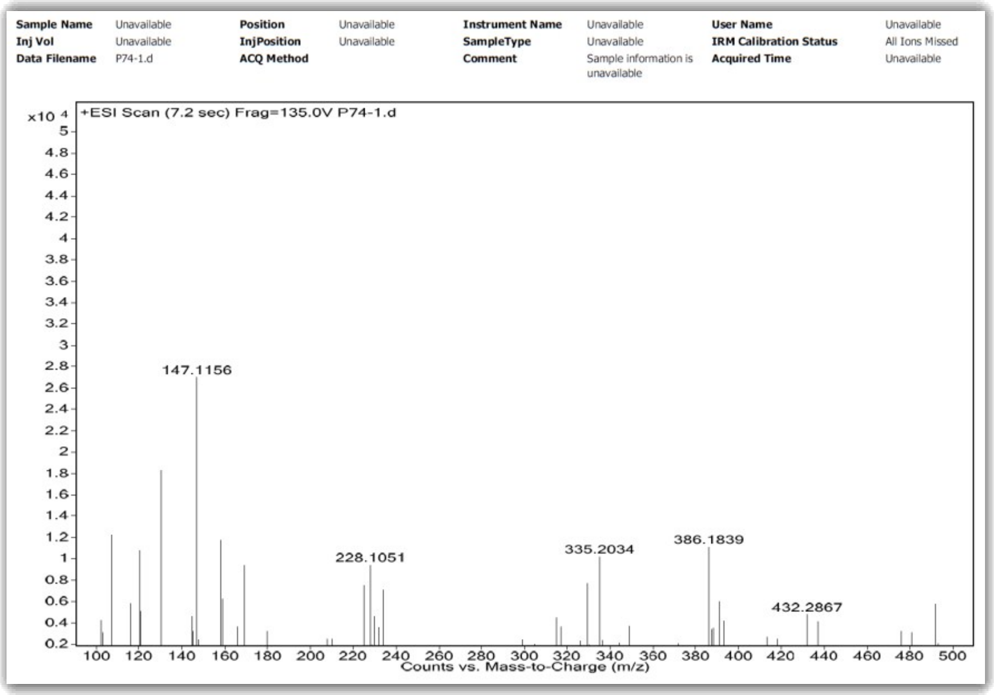

Mass Spectra of NIBD

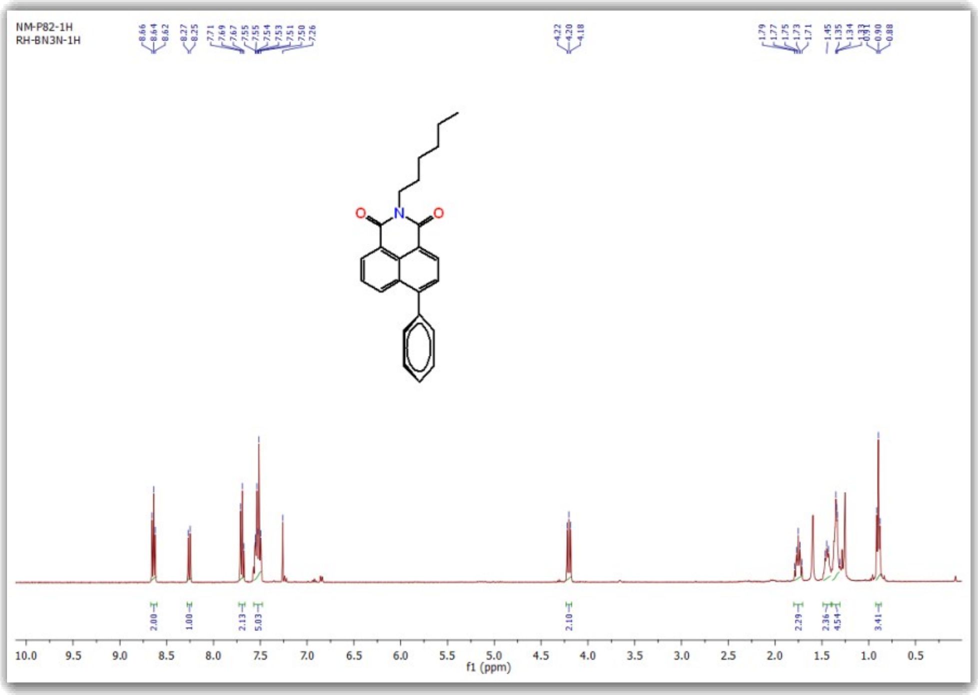

^{1}H NMR of NIB

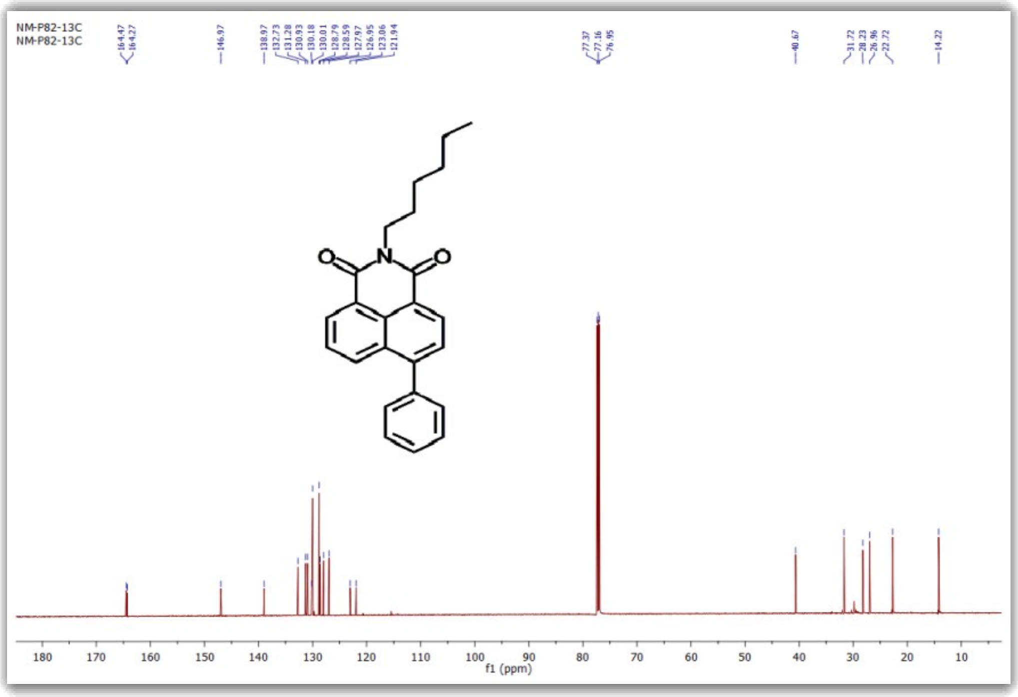

^{13}C NMR of NIB

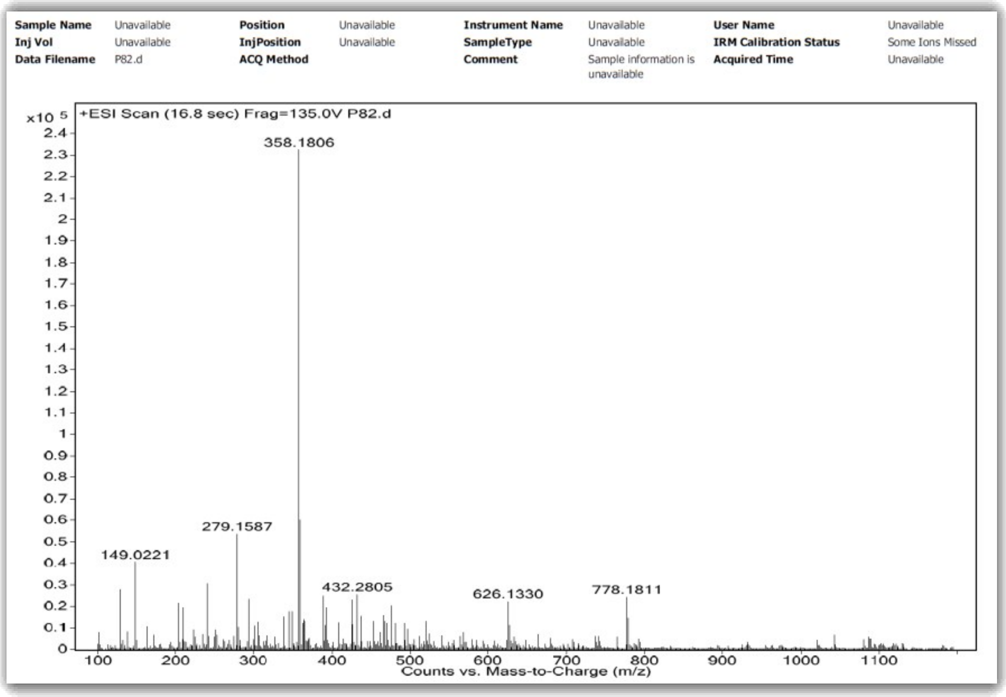

Mass Spectra of NIB

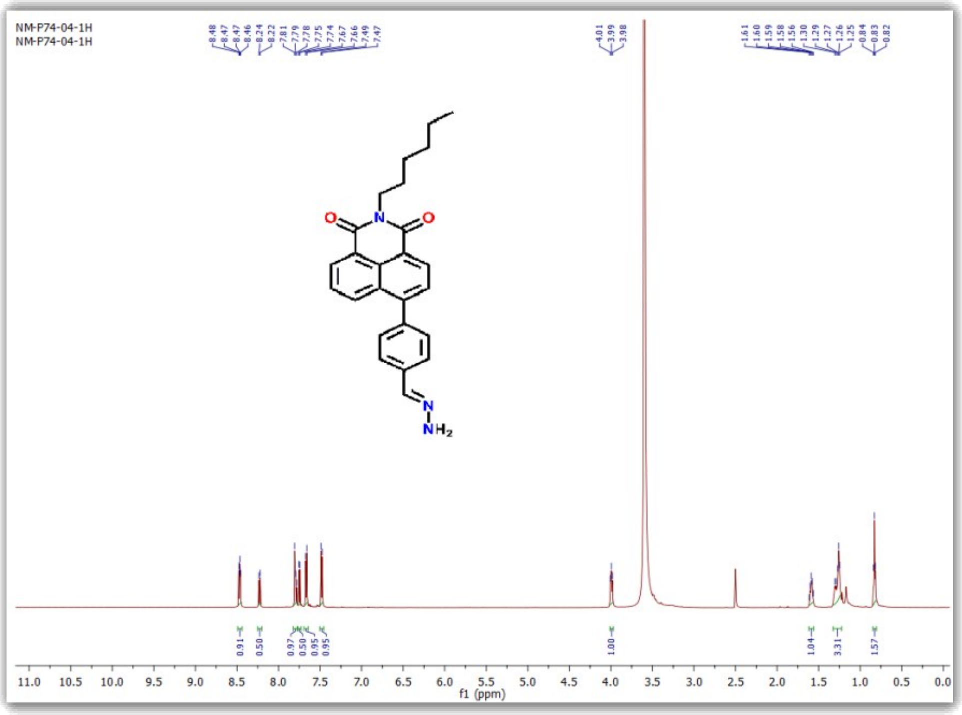

^{1}H NMR of NIBDH

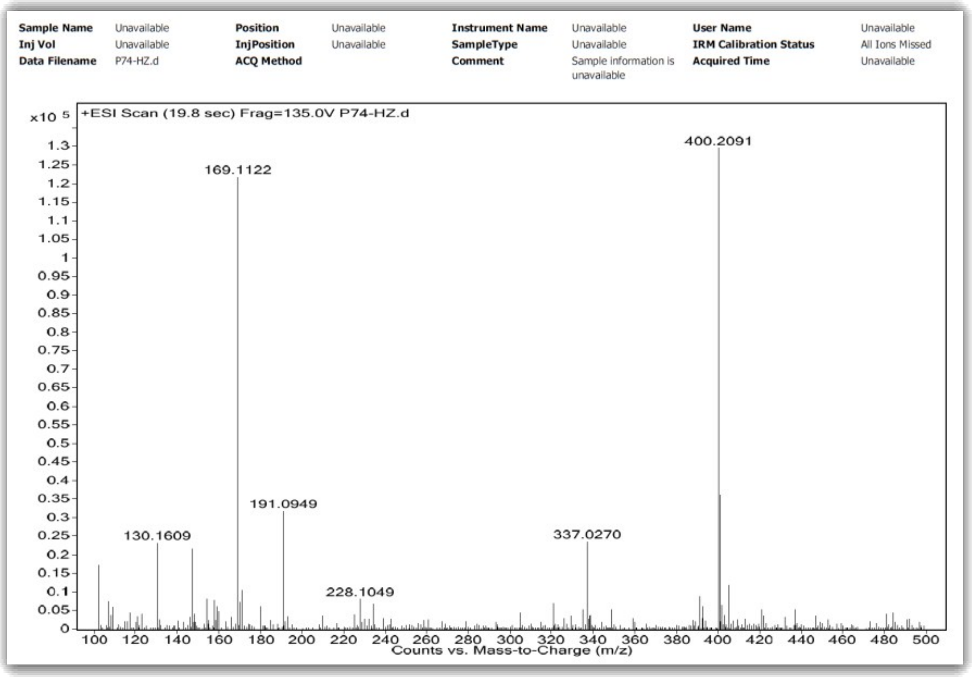

Mass Spectra of NIBDH

10. References

1. 1. R. Zhang, C.-J. Zhang, Z. Song, J. Liang, R. T. K. Kwok, B. Z. Tang and B. Liu, *J. Mater. Chem. C,* 2016, **4**, 2834-2842.
2. F. Ali, H. A. Anila, N. Taye, D. G. Mogare, S. Chattopadhyay and A. Das, *Chem. Commun.,* 2016, **52**, 6166-6169.
3. X. Dai, Z.-Y. Wang, Z.-F. Du, J.-Y. Miao and B.-X. Zhao, *Sensors and Actuators B,* 2016, **232**, 369–374.
4. R. Maji, A. K. Mahapatra, K. Maiti, S. Mondal, S. S. Ali, P. Sahoo, S. Mandal, M. R. Uddin, S. Goswami, C. K. Quahd and H.-K. Fun, *RSC Adv.,* 2016, **6**, 70855-70862.
5. J. Zhang, L. Ning, J. Liu, J. Wang, B. Yu, X. Liu, X. Yao, Z. Zhang and H Zhang, *Anal. Chem.,* 2015, **87**, 9101−9107.

6. J. Zhou, R. Shi, J. Liu, R. Wang, Y. Xu and X. Qian, *Org. Biomol. Chem.,* 2015, **13**, 5344-5348.

7. L. Cui, Z. Peng, C. Ji, J. Huang, D. Huang, J. Ma, S. Zhang, X. Qia**NIB** and Y. Xu, *Chem. Commun.,* 2014, **50**, 1485-1487.

8. X.-X. Zhao, J.-F. Zhang, W. Liu, S. Zhou, Z.-Q. Zhou, Y.-H. Xiao, G. Xi, J.-Y. Miao and B.-X. Zhao, *J. Mater. Chem. B,* 2014, **2**, 7344-7350.

9. L. Cui, C. Ji, Z. Peng, L. Zhong, C. Zhou, L. Yan, S. Qu, S. Zhang, C. Huang, X. Qian, and Y. Xu, *Anal. Chem.*, 2014, **86**, 4611−4617.

10. M. Sun, J. Guo, Q. Yang, N. Xiao and Y. Li, *J. Mater. Chem. B,* 2014, **2**, 1846-1851.

11. L. Xiao, J. Tu, S. Sun, Z. Pei, Y. Pei, Y. Pang and Y. Xu, *RSC Adv.,* 2014, **4**, 41807-41811.

12. M. H. Lee, B. Yoon, J. S. Kim and J. L. Sessler, *Chem. Sci.,* 2013, **4**, 4121-4126.

13. S. Goswami, S. Das, K. Aich, B. Pakhira, S. Panja, S. K. Mukherjee and S. Sarkar, *Org. Lett.,* 2013, **15**, 5412–5415.

14. C. Hu, W. Sun, J. Cao, P. Gao, J. Wang, J. Fan, F. Song, S. Sun and X. Peng, *Org. Lett.,* 2013, **15**, 4022–4025.

15. S. Goswami, S. Paula and A. Manna, *RSC Adv.,* 2013, **3**, 18872-18877.

16. M. G. Choi, J. Hwang, J. O. Moon, J. Sung and S.-K. Chang, *Org. Lett.,* 2011, **13**, 5260–5263.

17. M. G. Choi, J. O. Moon, J. Bae, J. W. Lee and S.-K. Chang, *Org. Biomol. Chem.,* 2013, **11**, 2961-2965.
